# Semantic Web Applications and Tools for Life Sciences, 2008 – Introduction

**DOI:** 10.1186/1471-2105-10-S10-S1

**Published:** 2009-10-01

**Authors:** Albert Burger, Paolo Romano, Adrian Paschke, Andrea Splendiani

**Affiliations:** 1 Heriot-Watt University and MRC Human Genetics Unit, Edinburgh, UK; 2 National Cancer Research Institute, Genova, Italy; 3 Free University Berlin, Berlin, Germany; 4 Rothamsted Research, Harpenden, UK

Semantic Web technologies play an increasingly important role in the integration of heterogeneous biomedical information as well as the generation of meaningful links between biological and clinical information resources, which are at the basis of both, translational and personalized, medicine. A critical component of the emergence of these technologies is the need to represent domain knowledge, e.g. biological and medical knowledge, in the systems that we build in order to facilitate not just the low level integration of resources, but to enable knowledge-driven analysis and reasoning across them. As is true for many other scientific disciplines, in spite of the potential benefits to be gained, we are still far from seeing a widespread adoption of the Semantic Web for the Life Sciences. Part of the problem is of course the large investment of effort needed to develop sufficiently formal representations of enough Life Science knowledge in the form of ontologies and other knowledge bases, but there are also issues with the maturity of the tools and applications that are currently available to those who want to develop and use Semantic Web applications and tools in the Life Sciences.

The purpose of the SWAT4LS workshops http://www.swat4ls.org is to create a space for discussions involving experts from the Computing as well as the Life Sciences, filling the gap between the possibilities and the practicalities of the Semantic Web in the Life Sciences domain. The first of these took place in Edinburgh on 28 November 2008 and at its heart was the question as to what is hindering a more rapid adoption of Semantic Web technologies in this domain. To reflect this, the panel discussion at the Edinburgh workshop was held under the heading of "If the Semantic Web is so good, why is everybody using OBO for ontologies and PERL for data integration?".

The original call for the SWAT4LS 2008 workshop resulted in the total of 40 submissions which were peer reviewed: 8 were accepted as full papers, 4 as poster papers and one as a demo paper. In addition, a number of posters were accepted. The workshop took place at the e-Science Institute in Edinburgh and was attended by about 80 participants. Following the actual workshop, all contributors to SWAT4LS'08 (papers as well as posters) were invited to submit extended versions of their contributions, resulting in the submission of 21 papers, which underwent another complete peer review cycle. Of these, 13 papers were selected and are included in this *BMC Bioinformatics* supplement.

We are delighted with the collection of high quality papers that are the result of this endeavor and are confident that the readers will find them helpful in their own work on Semantic Web technologies for the Life Sciences.

We would like to thank all who have contributed to the SWAT4LS 2008 workshop and this *BMC Bioinformatics* supplement. Specifically, we thank all the authors for their contributions, all the reviewers who served on the program committee for the original workshop call and then again for the submissions for this supplement, the invited speakers (Prof. Michael Krauthammer, Dr. M. Scott Marshall and Prof. Mark D. Wilkinson) and all the participants who made the workshop a memorable event. The support from the e-Science Institute in Edinburgh, which has hosted the workshop and covered some of the costs for our invited speakers, is also gratefully acknowledged. We also thank our sponsors: OMII-UK, LabAge SA, Leaf Bioscience and Textensor Limited for their support., Finally, we thank the staff at BMC Bioinformatics, specifically Ms. Isobel Peters, for their help with editing and processing this supplement.

Albert Burger, Paolo Romano, Adrian Paschke and Andrea Splendiani (SWAT4LS Supplement Editors and Co-chairs of SWAT4LS 2008)

